# Nabais Sá-de Vries Syndrome Type 1 in a Mexican Girl: A Case Report

**DOI:** 10.7759/cureus.107064

**Published:** 2026-04-14

**Authors:** Oscar Olivares-Huerta, Dulce María Castro-Coyotl, Israel Enrique Crisanto-López, Jonathan Cervantes-Larios, Renata Ochoa-Precoma, Blanca Frisia Morales-López, Itzel Alejandra Trejo-Toscano, Daniela Juárez-Melchor

**Affiliations:** 1 Department of Medical Genetics, General Hospital Zone No. 20, Mexican Social Security Institute, Puebla, MEX; 2 University Center for Health Sciences, University of Guadalajara, Guadalajara, MEX; 3 Department of Medical Genetics, Teleton Children's Rehabilitation and Inclusion Center, Puebla, MEX; 4 Department of Medicine, Meritorious Autonomous University of Puebla, Puebla, MEX; 5 Department of Pediatric Cardiology, General Hospital Zone No. 20, Mexican Social Security Institute, Puebla, MEX

**Keywords:** developmental disabilities, exome sequencing, failure to thrive, gain-of-function mutation, genetic heterogeneity, microcephaly, missense mutation, rare disease

## Abstract

Nabais Sá-de Vries syndrome (NSDVS) is a rare disease caused by a heterozygous mutation in the *SPOP* gene (17q21), which is involved in protein degradation via the ubiquitin-proteasome pathway. Different germline variants within this gene cause two distinct phenotypes (allelic heterogeneity) that affect multiple organs and systems (pleiotropy). These variants are clinically associated with intellectual disability, neurological disorders, and dysmorphic features that exhibit variable expressivity.

Here, we present the case of a five-year-old girl who was conceived through assisted reproductive technology. She is the second twin of a dichorionic dizygotic pregnancy. A medical genetic evaluation was initiated at one week of age due to congenital heart disease. She presents with a fronto-parieto-occipital hemangioma, downslanting palpebral fissures, synophrys, retroauricular pits, a depressed nasal bridge, anteverted nares, midface retrusion, a high palate, a micrognathia, an inguinal hernia, bilateral single transverse palmar creases, and a congenital melanocytic nevus. She currently has delayed neurodevelopment and language acquisition, as well as microcephaly, low weight and height, and normal hearing. Exome sequencing revealed a heterozygous missense variant: NM_001007228.2(*SPOP*):c.351G>T(p.Met117Ile). In silicotesting classifies this variant as likely pathogenic with protein gain of function, which confirms the diagnosis of NSDVS type 1.

This case report of a Mexican girl contributes to the expansion of the phenotypic spectrum of NSDVS and supports the implementation of improved multidisciplinary follow-up for affected patients.

## Introduction

Nabais Sá-de Vries syndrome (NSDVS) is a rare disease with a prevalence of less than one in a million inhabitants; it is characterized by intellectual impairment, neurological signs and symptoms, and variable dysmorphic features [1]. First described in 2020 by Nabais Sá and de Vries, two clinical entities were identified. The first presents intellectual impairment, motor and language delay, and congenital anomalies such as microcephaly, hypoacusia, and facial features including a small forehead, arched eyebrows, blepharophimosis, bulbous nasal tip, a smooth filtrum, micrognathia, and a pointed chin. In contrast, phenotype number 2 is characterized by relative macrocephaly, a broad and tall forehead, and hypotelorism. This indicates allelic heterogeneity of this gene. The second phenotype is also associated with systemic manifestations such as short stature and failure to thrive, as well as cardiovascular and endocrinological anomalies, epilepsy, and sleep disorders, demonstrating pleiotropy [2].

The *SPOP* gene (17q21 locus) encodes the speckle-type POZ protein and is responsible for NSDVS. This protein has three domains: an N-terminal MATH (meprin and tumor necrosis factor receptor-associated factor homology) domain, an intermediate BTB or POZ domain, and a C-terminal nuclear sequence [3]. It promotes the degradation of target proteins via the ubiquitin-proteasome pathway and plays a role in tumor suppression by destabilizing downstream oncoproteins [4,5]. It has been proposed that gain-of-function variants, which increase gene expression, have a dominant-negative effect, minimizing the concentration of bromodomain and extraterminal (BET) proteins (BRD2, BRD3, and BRD4) and causing NSDVS type 1 (OMIM: 618828). Alternatively, the over-regulation of BET proteins causes a loss of function, resulting in the development of NSDVS type 2 phenotype (OMIM: 618829) [2].

We present herein the phenotypic characteristics and manifestations of a missense mutation in the *SPOP* gene in a female patient, thereby expanding the phenotypic spectrum of the syndrome. To date, the global literature has not reported any other cases of this mutation alongside 12 other cases associated with the *SPOP* gene.

## Case presentation

**Figure 1:**
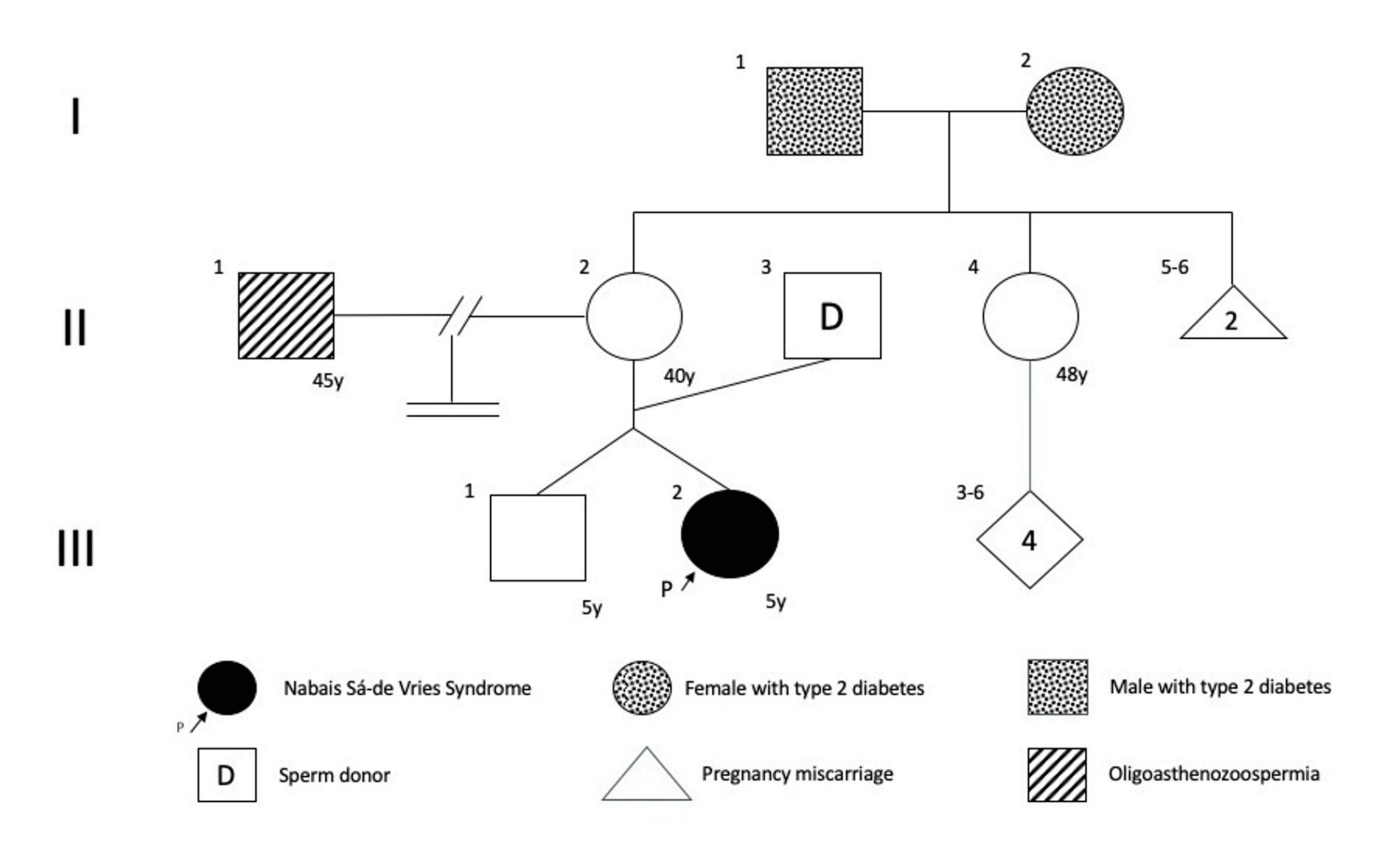
Pedigree. This image shows a pedigree of a family spanning three generations (I, II, and III), with each individual represented by an Arabic numeral (1, 2, 3, etc.). The blank square (males), circle (females), and rhombus (a group of individuals) represent healthy individuals. A triangle represents miscarriage during pregnancy. The proband (III2), the outcome of a twin pregnancy achieved through assisted reproductive technology, is the only individual affected with NSDVS within her family. This finding suggests a de novo nature of the mutation. NSDVS: Nabais Sá-de Vries syndrome

We present the case of a five-year-old girl, daughter of a couple who had experienced infertility issues for four years due to uterine alterations and oligoasthenozoospermia. They had undergone multiple attempts to conceive using assisted reproductive technology with a sperm donor (see Figure 1). A dichorionic dizygotic pregnancy was obtained through in vitro fertilization product; on a third-trimester ultrasound, intrauterine retardation growth was detected in our patient. Delivered at 35 weeks of gestation via cesarean section, a healthy male (twin A) and female (twin B) exhibited spontaneous crying and effective respiratory effort at birth.

During the first postnatal days, twin A had an adequate clinical course; in contrast, twin B (proband) developed respiratory distress, prompting a transthoracic ultrasound. Findings included a patent ductus arteriosus, a patent foramen ovale, and pulmonary hypertension, which added to minor dysmorphic features and warranted a medical genetic evaluation.

Her initial physical examination showed a fronto-parieto-occipital hemangioma, downslanting palpebral fissures, mild synophrys, retroauricular pits, a depressed nasal bridge, anteverted nares, midface retrusion, a high-arched palate, micrognathia, and a protruding tongue; the thorax and abdomen were normal; and external genitalia exhibited a typical female phenotype. Additional findings included a left inguinal hernia, bilateral single transverse palmar creases, and a congenital melanocytic nevus. Given the history of infertility and assisted reproductive technology, an epigenetic defect or structural chromosomal anomaly was considered. Therefore, cytogenetic testing was performed, reporting a 46,XX karyotype.

**Figure 2:**
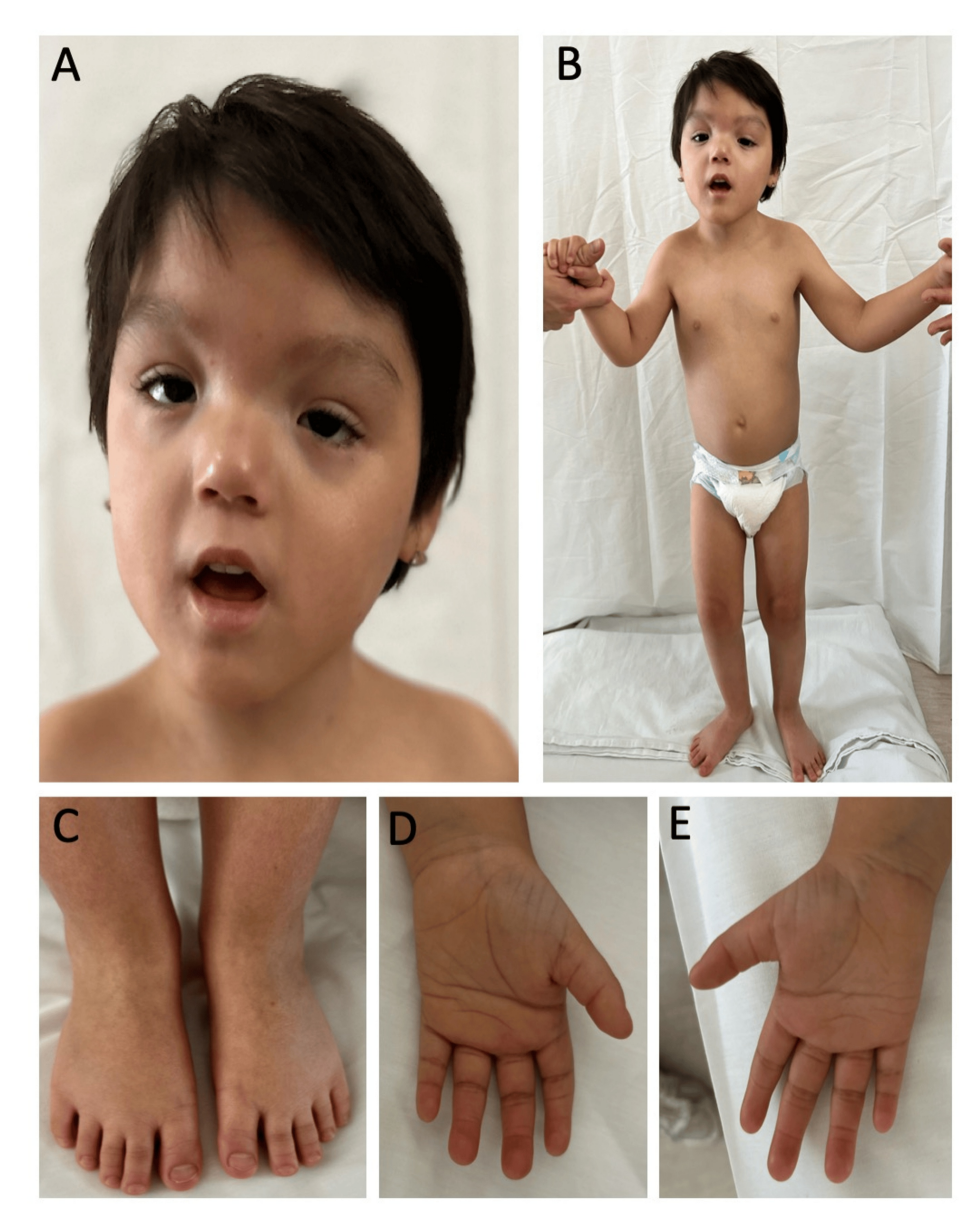
Physical examination. (A) Frontal view of the patient's face showing facial asymmetry with downslanting palpebral fissures, a wide nasal bridge, bulbous nasal tip, and a pointed chin. (B) Full-body frontal view of the patient showing thorax asymmetry and widely spaced nipples. (C) Dorsal view of the feet, bilateral cutaneous syndactyly of T2-T3, and shortening of left foot T4-T5 are appreciated. (D-E) Palmar view of the hands with tapered fingers and bilateral transverse palmar creases. The patient's mother consented to have the child's identity revealed, and a written and signed consent was provided to the journal.

Regarding growth and development, the patient has a severe delay in achieving developmental milestones (Table 1). Her most recent physical examination is summarized in Figure 2, with short stature, microcephaly, and facial dysmorphisms as notable traits.

**Table 1:** Developmental milestones.

Developmental milestones	Time of accomplishment	Normal milestones accomplishment
Lift head	9 months	1 month
Sit (no support)	3 years	5-7 months
Stand holding on	3 years and 6 months	6-9 months
Walk well	Unaccomplished	11-15 months
Sphincter control	Unaccomplished	Urinary continence 18 months to 5 years; fecal continence <4 years
Language	Single syllables	Equal to 4-7 months

In addition, due to cardiac history and neurological findings, whole exome sequencing was performed, identifying a variant of uncertain significance in the *SPOP* gene, NM_001007228.2:c.351G>T, which results in a methionine-to-isoleucine substitution at position 117. This variant is consistent with NSDVS.

## Discussion

NSDVS is primarily associated with missense and nonsense variants and follows an autosomal dominant inheritance pattern. To date, 12 cases of NSDVS have been reported worldwide, with the patient described here representing the first identified case in the Mexican population and the sixth associated with a gain-of-function variant [1-3,6-8]. Currently, approximately 10 missense variants have been correlated to the NSDVS phenotype types 1 and 2 worldwide, and one nonsense variant has been correlated with both phenotypes (Table 2).

**Table 2:** Genetic variants reported worldwide associated with NSDVS. NSDVS: Nabais Sá-de Vries syndrome

*SPOP* gene variant	Author(s)	Country	Number of individuals
NSDVS type 1			
c.351G>T (p.Met117Ile)	This case report	Mexico	1
c.362G>A (p.Arg121Gln)	Nabais Sá et al., 2020 [2]	Netherlands	1
	Zhang et al., 2022 [7]	USA	1
c.361C>T (p.Arg121Trp)	Galli et al., 2025 [1]	Italy	1
c.430G>A (p.Asp144Asn)	Nabais Sá et al., 2020 [2]	Netherlands	1
c.977A>G (p.Asn326Ser)	Alam et al., 2024 [6]	Saudi Arabia	1
NSDVS type 2			
c.67T>C (p.Cys23Arg)	Hu et al., 2022 [3]	China	1
c.73A>G (p.Thr25Ala)	Nabais Sá et al., 2020 [2]	Netherlands	1
c.248A>G (p.Tyr83Cys)	Nabais Sá et al., 2020 [2]	Netherlands	1
c.395G>T (p.Gly132Val)	Nabais Sá et al., 2020 [2]	Netherlands	1
c.412C>T (p.Arg138Cys)	Nabais Sá et al., 2020 [2]	Netherlands	2
NSDVS type 1/2			
c.1059C>A (p.Tyr353*)	Law and Lam, 2022 [8]	Hong Kong Special Administrative Region	1

The *SPOP* gene variant NM_001007228.2(*SPOP*):c.351G>T(p.Met117Ile) results in a missense mutation in the corresponding protein. According to the criteria for variant classification by the American College of Medical Genetics and Genomics and the Association for Molecular Pathology [9], the variant's allelic frequency is reported as extremely low in the Genome Aggregation Database (gnomAD) population databases, and it is classified as pathogenic moderately (PM2). Assessed in silico analyses classify this variant as pathogenic moderate (PP3), suggesting a gain-of-function effect that reduces BET protein concentrations. No functional well-established assays are available for this variant. Unfortunately, no segregation assessment was made. A de novo status is assumed (PM6) due to the patient's pedigree. And lastly, this variant involves the substitution of a methionine (a sulphur-containing amino acid) for isoleucine (a highly hydrophobic amino acid) in the MATH domain. This affects the ability of the critical functional domain to recognize substrates, resulting in moderately pathogenic (PM1) criterion. Taken together with the patient's clinical phenotype, the three moderate evidences (PM1, PM2, and PM6) and the one supporting evidence (PP3) make us advocate that this variant be reclassified.

Unlike what has been previously reported, our patient does not have hypoacusia, which is a defining characteristic of NSDVS type 1. In addition, she exhibits low height, delayed bone growth, and severe developmental delay according to the Denver II developmental milestones [10]. Besides, congenital cardiovascular anomalies, mesial temporal sclerosis, subependymoma, dermatological anomalies, and craniofacial dysmorphisms such as dacryostenosis have not been previously described in association with NSDVS type 1. The genetic heterogeneity and pathogenicity observed in *SPOP* gene variants are due to polarity effects and changes in volume caused by amino acids [7,8]. This influences the affinity between the MATH domain and the protein substrate. However, it is still unclear how specific germinal gene variants and clinical manifestations are correlated. This case report allows us to expand on the phenotypic findings of NSDVS type 1 (Table 3).

**Table 3:** Clinical findings of patients with Nabais Sá-de Vries syndrome type 1. NR: non-reported; SD: standard deviation; -: normal; MATH: meprin and tumor necrosis factor receptor-associated factor homology

Author	Nabais Sá et al., 2020 [2]		Law and Lam, 2022 [8]	Zhang et al., 2022 [7]	Alam et al., 2024 [6]	Galli et al., 2025 [1]	Patient reported
Variants identified in individuals	c.362G>A (p.Arg121Gln)	c.430G>A (p.Asp144Asn)	c.1059C>A (p.Tyr353*)	c.362G>A (p.Arg121Gln)	c.977A>G (p.Asn326Ser)	c.361C>T (p.Arg121Trp)	c.351G>T (p.Met117Ile)
								Mutation type	Missense	Missense	Nonsense	Missense	Missense	Missense	Missense
Domain	MATH	MATH	C-terminal	MATH	C-terminal	MATH	MATH
Exon	5	5	10	5	9	5	4
Growth and development							
Age	4y7m	10y	1y2m	4y6m	4y	11y	5y
Height	104.5 cm (50th percentile)	77 cm (10-25th percentile)	10-25th percentile	103 cm (25-50th percentile)	109 cm (90th percentile)	NR	82 cm (SD -6.1)
Weight	15.3 kg (10-25th percentile)	8.8 kg (SD -2.3)	<3rd percentile	15.2 kg (3-10th percentile)	16 kg (50th percentile)	NR	12 kg (SD -3.79)
Head circumference	44 cm (SD -4)	40.5 cm (SD -5)	<3rd percentile	44 cm (SD -3)	46 cm (<3rd percentile)	NR	43 cm (SD -7.55)
Perinatal history							
Previous pregnancy loss/infertility	NR	NR	-	NR	Yes, two previous pregnancy losses	NR	Infertility
Assisted reproduction techniques	NR	NR	NR	NR	No	NR	IVF with a sperm donor
Assigned sex at birth	Female	Male	Female	Male	Female	Female	Female
Gestational age at birth	40.3 weeks	39 weeks	Full-term	40 weeks	NR	39 weeks	35 weeks
Length at birth	47 cm (10-25th percentile)	NR	NR	48.2 cm	48.2 cm (25th percentile)	47 cm (10th percentile)	NR
								Weight at birth	3,033 g (10-25th percentile)	2,409 g (3rd percentile)	NR	3,200 g	2,800 g (25th percentile)	2,780 g (15th percentile)	NR
Head circumference at birth	32 cm (3rd percentile)	Microcephaly reported	NR	Microcephaly reported	33 cm (10th percentile)	32 cm (3rd percentile)	NR
Fetal ultrasound	Shortened long bones (SD < -3)	-	NR	NR	NR	NR	Twin pregnancy
							Intrauterine growth restriction
Congenital anomalies	Congenital microcephaly	Congenital microcephaly	NR	NR	Congenital microcephaly	NR	Patent foramen ovale and patent ductus arteriosus
Additional findings	Jaundice requiring phototherapy	Jaundice, respiratory distress requiring tracheostomy	NR	Jaundice requiring phototherapy, respiratory distress	Jaundice requiring phototherapy, respiratory distress	Mother's gestational diabetes, feeding problems	Prematurity, respiratory distress, twin B
Motor skill development							
Psychomotor delay	Yes	Yes	Yes	Yes	NR	Yes	Yes
Walk well	1y7m	3y	NR	1y7m	NR	1y4m	Assisted walking
Language delay	Yes	Yes	Yes	Yes	Yes	-	Yes
Spoken language	60 words at 2y10m	Vocalize at 6y	NR	Vocalize at 1y4m	NR	1y	Monosyllables only
Intellectual disability	Global neurodevelopmental delay	Severe intellectual disability	Mild intellectual disability	Global neurodevelopmental delay	NR	NR	Global neurodevelopmental delay
Neuropsychiatric abnormalities							
Epilepsy	-	NR	-	Two seizure-like episodes	Two seizure-like episodes	NR	-
Neurological symptoms	Hypertonia	NR	Hypotonia	Mild hypertonia, hyperactive deep tendon reflexes	NR	NR	Mild hypotonia
Central nervous system abnormalities	5a: simplified convolutions	NR	-	12m: -	-	4y: simplified secondary and tertiary convolutions	2y: mesial temporal sclerosis, subependymoma
Behavior disorder	Stranger anxiety	Self-harm	Stranger anxiety	Poor frustration tolerance (aggressiveness)	Hyperactivity, deficient interaction and understanding	-	-
Craniofacial dysmorphisms							
Cranium/face	Brachycephaly, prominent glabella	Narrow forehead, low anterior hairline	Narrow forehead	Brachycephaly, triangular face	Long and triangular face	Round face, narrow forehead	Apparent dolichocephaly, high anterior hairline, facial asymmetry, triangular face, midface hypoplasia
Ears	Dysplastic simple ears with a thickened helix	NR	Low-set ears	Long low-set ears, overfolded helix	Large ears with a prominent antitragus	Low-set ears	Small ears with prominent antitragus, retroauricular pits
Eyes	Highly arched eyebrows, underdevelopment of the supraorbital ridge, short and downslanted palpebral fissures, deeply set eyes	Highly arched eyebrows, synophrys, long eyelashes, epicanthus, telecanthus, short palpebral fissures	Epicanthus, upslanted palpebral fissures, long eyelashes	Downslanted palpebral fissures, bilateral epicanthus, enophthalmos, esotropia, long eyelashes	Highly arched eyebrows, widely spaced eyes	Highly arched and laterally sparse eyebrows, deeply set eyes, long palpebral fissures, blepharophimosis	Highly arched and laterally sparse eyebrows, synophrys, epicanthus and epicanthus inversus bilaterally, widely spaced eyes, blepharophimosis, downslanted palpebral fissures, bilateral dacryostenosis
								Nose	Prominent nasal bridge, wide and bulbous nasal tip, underdeveloped alae nasi	Short nose with a broad and depressed nasal bridge, wide and bulbous nasal tip	Depressed nasal bridge	Small nose with a depressed nasal bridge	Wide and prominent nasal bridge, wide and bulbous nasal tip	Short and prominent columella, bulbous nasal tip, hypoplastic and anteverted nares	Wide and depressed nasal bridge, wide nasal ridge and bulbous nasal tip, high insertion of the columella and anteverted nares
								Mouth	Flat philtrum	Flat philtrum	NR	-	-	Flat philtrum, thin upper lip, enlarged upper central incisors, hypoplastic upper lateral incisors	Small mouth, flat philtrum, depressed labial commissures, high and narrow palate, mild protruding tongue
Chin	Pointed chin	Pointed chin	NR	Micro-/retrognathia	Pointed chin and micrognathia	Pointed chin	Pointed chin, micrognathia
Limb abnormalities							
Upper	Fifth finger clinodactyly	NR	Clinodactyly, tapered fingers	NR	NR	NR	Tapered fingers, bilateral transverse palmar crease
Lower	NR	NR	NR	NR	NR	NR	Bilateral cutaneous syndactyly of T2-T3, shortening of the left foot T4-T5; bilateral flatfoot
Other phenotypical abnormalities							
Hearing impairment	Neurosensorial hearing loss	Bilateral hearing loss	-	Bilateral neurosensorial hearing loss	NR	NR	-
Vision impairment	NR	Bilateral optic nerve hypoplasia	-	Bilateral hyperopia with astigmatism	NR	Hypermetropia + astigmatism, retinal hypopigmentation. Oculomotor dysfunction (horizontal nystagmus, alternating esotropia, deficit bilateral abduction impairment, saccadic movements, and interrupted tracking)	-
								Cardiovascular	NR	NR	-	NR	NR	-	Reported at congenital anomalies
Respiratory	NR	Tracheostomy at 3m	-	NR	NR	NR	-
Gastrointestinal	NR	Gastrostomy at 3m	Laryngomalacia	Oropharyngeal dysphagia, chronic constipation	NR	NR	Laryngomalacia
Urogenital	NR	Bilateral vesicoureteral reflux	NR	Secondary enuresis	NR	NR	Left inguinal hernia
Dermatological	NR	NR	NR	NR	NR	NR	Fronto-parieto-occipital hemangioma, congenital melanocytic nevi, zygomatic hyperpigmented spot
								Endocrinological	NR	NR	-	Normal bone age, failure to thrive	NR	-	Bone age of 3y at 4y8m (Greulich and Pyle)
Sleep disturbance	NR	NR	Sleep myoclonus	Severe obstructive sleep apnea, bruxism, hypoventilation, abnormal postures	NR	NR	-
Others	Sacral dimple	NR	-	Coccygeal sebaceous cyst	β-Thalassemia	NR	Thorax asymmetry, widely spaced nipple, mild scoliosis

In contrast, somatic cell mutations cause the occurrence and progression of gastric, colorectal, and prostate cancer [11]. This is due to the dysregulation of the function as an adaptor protein of the substrate of the complex ligase-E3 ubiquitin, which mediates the ubiquitinization and proteasomal degradation of multiple substrates, such as BET proteins, androgen receptor (AR), c-Myc, PD-L1, and transcription factors from the Hedgehog pathway [12].

The BET proteins participate in interactions between proteins, as well as between proteins and nucleic acids. This gives them the function of epigenetic interpreters [13]. Although the proband was obtained through assisted reproductive technology, there is currently no evidence to suggest a significant association between this technology and the occurrence of de novo mutations [14]. This does not correlate with *SPOP* somatic gene alterations that predispose individuals to oncoprotein dysregulation. However, cancerous cases have not yet been reported in NSDVS.

At present, no guidelines have been issued for follow-up. Consequently, a multidisciplinary team is required for comprehensive care, including specialists in neurology, cardiology, ophthalmology, and otorhinolaryngology, among others, owing to the fact that multiple organs and systems are affected. Such coordinated follow-up is essential to monitor clinical manifestations and to prevent or manage potential complications. In addition, ongoing evaluation by clinical geneticists is crucial to assess patient outcomes and to ensure the early identification of unreported signs and symptoms.

## Conclusions

NSDVS is a recently described rare disease. It is caused by *SPOP* gene variants located on the 17q21 locus. This gene promotes protein degradation via the ubiquitin proteasome pathway and plays an important role in tumor suppression. However, there is no physiopathological explanation for the phenotypic changes associated with germinal variants. 

NSDVS exhibits allelic heterogeneity, pleiotropy, and variable expressivity, resulting in two clinically distinct phenotypes. This makes genetic testing and a physical examination essential for diagnosis. This case report of a Mexican girl contributes to the expansion of the phenotypic spectrum of NSDVS and supports the implementation of improved multidisciplinary follow-up for affected patients.
